# Editorial: Immunological characteristics and novel therapeutic targets for metastatic gastrointestinal tumors

**DOI:** 10.3389/fimmu.2024.1476405

**Published:** 2024-08-28

**Authors:** Hao Chi, Lai Jiang, Xuancheng Zhou, Gang Huang, Honghao Luo, Ke Xu, Xiaosong Li

**Affiliations:** ^1^ Clinical Medical College, Southwest Medical University, Luzhou, China; ^2^ Department of Radiology, Xichong People’s Hospital, Nanchong, China; ^3^ Department of Oncology, Chongqing General Hospital, Chongqing University, Chongqing, China; ^4^ Clinical Molecular Medicine Testing Center, The First Affiliated Hospital, Chongqing Medical University, Chongqing, China; ^5^ Western (Chongqing) Collaborative Innovation Center for Intelligent Diagnostics and Digital Medicine, Chongqing National Biological Industry Base, Chongqing, China

**Keywords:** metastatic gastrointestinal tumor, immunological characteristic, immune tolerance, biomarker, prognosis

## 1 Introduction

The challenges of gastrointestinal malignant tumors have always been present in the journey of discovery in modern medicine. Despite significant advances in diagnostic techniques and surgical approaches, the prognosis of advanced gastrointestinal malignancies remains challenging. Metastasis and recurrence of these tumors not only pose a threat to the patient’s life, but also put clinical treatment strategies to a severe test (1). Tumor metastasis is a multistep and highly complex biological process, the core of which lies in the interactions between tumor cells and the tumor microenvironment, especially the mechanism by which tumor cells evade the host immune system attack (2). In recent years, the concept of immune tolerance has received increasing attention in the field of oncology, which is not only related to the survival and proliferation of tumor cells, but also closely associated with the remodeling of the tumor microenvironment (3).

In this Research Topic, we aim to delve into the latest advances in the treatment of gastrointestinal malignant tumors, especially in the identification of biomarkers, the analysis of immune tolerance mechanisms, and the innovation of therapeutic strategies. We focus on how to translate these findings into practical clinical applications, with a view to providing more effective and personalized treatment options for patients.

## 2 Biomarkers and molecular mechanisms

In exploring the complex biology of gastrointestinal tumors, the identification of biomarkers and the resolution of molecular mechanisms are of inestimable value in achieving precision medicine (4, 5). The studies assembled in this review delve into the important role of biomarkers in tumor development and reveal the functions of key molecules in the disease process, and they provide new perspectives for our understanding of gastrointestinal tumors at the molecular level. A study by Fan et al. revealed the expression pattern of EVA1A in colorectal cancer and its strong association with patient prognosis. EVA1A expression was found to be significantly upregulated in CRC tissues and significantly correlated with patient age, tumor metastasis and stage, and CA199 markers. This study not only provides new biomarkers for prognostic assessment of CRC, but also potential molecular targets for future targeted therapies. By combining single-cell transcriptomics and conventional bulk sequencing technologies, Yang et al.’s research team has deeply dissected the central role of PAK2 in pancreatic cancer liver metastasis and revealed its molecular mechanism of influencing tumor cell behavior through the TGF-β signaling pathway, which provides a new molecular perspective for the therapeutic strategy of pancreatic cancer. The study by Fan et al. explored the multiple roles of KHDRBS1 in hepatocellular carcinoma, including its effects on cell proliferation, migration, and the immune microenvironment, further emphasizing its potential as a prognostic biomarker and therapeutic target. These studies not only deepen our understanding of the molecular mechanisms of gastrointestinal tumors, but also provide new strategies and directions for future diagnosis and treatment.

## 3 Immune tolerance and therapeutic strategies

The phenomenon of immune tolerance in metastatic gastrointestinal tumors is a complex and critical obstacle in the tumor microenvironment that limits the ability of the immune system to recognize and remove tumor cells. Overcoming this obstacle is an important way to increase the efficiency of immunotherapy and improve patient prognosis. We have brought together two in-depth review articles that not only comprehensively analyze the molecular mechanisms leading to immune tolerance, but also explore possible interventions. These reviews provide a comprehensive understanding of the mechanisms of immune escape in gastrointestinal tumors and provide a scientific basis for the development of new therapeutic approaches. Gan et al. provide insight into the molecular mechanisms and signaling pathways that lead to immune tolerance in metastatic gastrointestinal tumors and discuss possible strategies to overcome these immune tolerance states. The study summarized several signaling pathways associated with immune escape in gastrointestinal tumors, including PI3K/AKT, JAKs-STAT3, NF-κB, TGF-β/Smad, Notch, PD-1/PD-L1, and Wnt-β-catenin-IL-10 signaling axis. Also, the article highlights the role of some key molecules such as IDO1, HLA-G/E, GARP, Clever-1, IRF8/OPN, TIM-3, CEACAM1, Cdc42, and Caspases-1 and -12 in immune tolerance. The discovery of these molecules and signaling pathways provides a theoretical basis for the development of new immunotherapeutic strategies and helps to design therapeutic approaches targeting specific immune escape mechanisms, with the aim of improving the therapeutic efficacy and survival of patients with metastatic gastrointestinal tumors. He et al. delved into immune interactions during CRC metastasis and attempted to decode the role of immune cells and molecules in CRC metastasis. The focus is on analyzing the immune escape mechanisms of CRC cells, such as the suppression of immune cells and molecules through the secretion of small extracellular vesicles (sEVs), up-regulation of PD-L1 expression, and the role of CTLA-4. The article highlights the emerging field of immunotherapy in the treatment of CRC, particularly in terms of therapies targeting immune checkpoints, and discusses how immune checkpoint inhibitors can be combined with other therapeutic approaches to improve treatment outcomes. These studies provide a deeper understanding of the immune microenvironment of gastrointestinal tumors and provide new ideas and directions for developing more effective immunotherapy strategies and personalized medicine.

## 4 Conclusion and outlook

In this Research Topic, we delve into a series of innovative research advances in the field of gastrointestinal malignancy treatment. These studies not only provide valuable insights into the tumor microenvironment, identification of biomarkers, and mechanisms of immune tolerance, but also point the way to future therapeutic strategies. Although these findings are theoretically important, further clinical trials and mechanistic studies are needed to realize their clinical applications. In conclusion, we have gained a more comprehensive understanding of the treatment of gastrointestinal malignant tumors through the in-depth discussion of this Research Topic. We expect that these research results will be translated into practical clinical applications in the future, bringing new hope and a brighter future for patients.
